# Supplementary material for: Identification of Conservation Priority Areas and a Protection Network for the Siberian Musk Deer (Moschus moschiferus L.) in Northeast China
Source: Animals (Basel). 2022 Jan 21;12(3):260. doi: 10.3390/ani12030260 (PMC8833384; doi:10.3390/ani12030260)
Supplement: Supplementary file 1 [file animals-12-00260-s001.zip › animals-1520134-supplementary.pdf]

Table S1. Records from literatures

ID	References
Fauna surveys	
1	Rare and Endangered Wild Animals and Plants in Heihe
Nature reserve scientific surveys	
1	Inner Mongolia Hanma National Nature Reserve
2	Heilongjiang Dazhan River Wetland National Nature Reserve
3	Heilongjiang Gongbila River National Nature Reserve
4	Heilongjiang Liangshui National Nature Reserve
5	Heilongjiang Cuibei Wetland National Nature Reserve
6	Heilongjiang Fenglin National Nature Reserve
7	Heilongjiang Hongxing Wetland National Nature Reserve
8	Heilongjiang Maolanggou National Nature Reserve
9	Heilongjiang Wuyiling National Nature Reserve
10	Heilongjiang Taipinggou National Nature Reserve
11	Heilongjiang Xinqing White-headed Crane National Nature Reserve
12	Heilongjiang Laoyeling National Nature Reserve
13	Heilongjiang Xiaobeihu National Nature Reserve
14	Heilongjiang Daxiagu National Nature Reserve
Scientific research	
1	Selection of musk deer winter habitat in Huangnihe Nature Reserve [1]
2	The Monitoring Research of the Amur Tiger in China by Using a Far Infrared Automatic Camera [2]
3	Analysis of the Law of on Population Quantity Changes of the Wild Animals *Martes zibellina*, *Moschus moschiferus*, *Tetrao parvirostris* in Ling Feng Nature Reserve [3]
4	Comparison of Home Range of Siberian Musk Deer and Coral in Winter [4]
5	Status and administrative measures of Moschus moschiferus resources in Liaoning province [5]
6	Resources Situation and Distribution of Moschus moschiferus in Changbai Mountains in Jilin Province [6]

Table S2. Protected areas within the potential distribution of *M. moschiferus*

ID	Name	Biogeographic realm	Area (ha)
1	Heilongjiang Beijicun National Nature Reserve	Greater Khingan Mountains zone	137553

ID	Name	Biogeographic realm	Area (ha)
2	Heilongjiang Lingfeng National Nature Reserve	Greater Khingan Mountains zone	68373
3	Heilongjiang Nanweng River National Nature Reserve	Greater Khingan Mountains zone	229523
4	Heilongjiang Huzhong National Nature Reserve	Greater Khingan Mountains zone	167213
5	Heilongjiang Pan National Nature Reserve	Greater Khingan Mountains zone	55074
6	Heilongjiang Shuanghe National Nature Reserve	Greater Khingan Mountains zone	88849
7	Inner Mongolia Bila River National Nature Reserve	Greater Khingan Mountains zone	56604
8	Inner Mongolia Hanma National Nature Reserve	Greater Khingan Mountains zone	107348
9	Inner Mongolia Erguna National Nature Reserve	Greater Khingan Mountains zone	124527
10	Heilongjiang Dazhan River Wetland National Nature Reserve	Lesser Khingan Mountains zone	211618
11	Heilongjiang Gongbila River National Nature Reserve	Lesser Khingan Mountains zone	47983
12	Heilongjiang Shengshan National Nature Reserve	Lesser Khingan Mountains zone	60000
13	Heilongjiang Wuyiling National Nature Reserve	Lesser Khingan Mountains zone	43824
14	Heilongjiang Xinqing White-headed Crane National Nature Reserve	Lesser Khingan Mountains zone	62567
15	Heilongjiang Cuibei Wetland National Nature Reserve	Lesser Khingan Mountains zone	27730
16	Heilongjiang Hongxing Wetland National Nature Reserve	Lesser Khingan Mountains zone	111995
17	Heilongjiang Uma River Sable National Nature Reserve	Lesser Khingan Mountains zone	20949
18	Heilongjiang Longxiang National Nature Reserve	Lesser Khingan Mountains zone	31355
19	Heilongjiang Liangshui National Nature Reserve	Lesser Khingan Mountains zone	12133
20	Heilongjiang Liangshui National Nature Reserve	Lesser Khingan Mountains zone	60687
21	Heilongjiang Maolanggou National Nature Reserve	Lesser Khingan Mountains zone	35868
22	Heilongjiang Pingdingshan National Nature Reserve	Lesser Khingan Mountains zone	20241
23	Heilongjiang Grand Canyon National Nature Reserve	Lesser Khingan Mountains zone	24998
24	Heilongjiang Xilian River National Nature Reserve	Lesser Khingan Mountains zone	21570
25	Heilongjiang Taipinggou National Nature Reserve	Lesser Khingan Mountains zone	22199
26	Heilongjiang Xiaobeihu National Nature Reserve	Changbai Mountains zone	20834
27	Liaoning Baishilazi National Nature Reserve	Changbai Mountains zone	7467
28	Liaoning Laotuziding National Nature Reserve	Changbai Mountains zone	15219
29	Jilin Baishan Musk deer National Nature Reserve	Changbai Mountains zone	21995
30	Jilin Huangnihe National Nature Reserve	Changbai Mountains zone	41583
31	Jilin Ji 'an National Nature Reserve	Changbai Mountains zone	13821.6
32	Jilin Songhua River Sanhu National Nature Reserve	Changbai Mountains zone	115253

ID	Name	Biogeographic realm	Area (ha)
33	Jilin Toudao Upper Songhua River National Nature Reserve	Changbai Mountains zone	13350
34	Jilin Yalu River upstream National Nature Reserve	Changbai Mountains zone	20306
35	Jilin Tianfozhi Mountain National Nature Reserve	Changbai Mountains zone	77317
36	Jilin Tonghua Shihu National Nature Reserve	Changbai Mountains zone	15200
37	Jilin Yuanchi Wetland National Nature Reserve	Changbai Mountains zone	17377
38	Jilin Zengfengling National Nature Reserve	Changbai Mountains zone	17386
39	Jilin Changbai Mountain National Nature Reserve	Changbai Mountains zone	196465
40	Northeast Tiger and Leopard National Park	Changbai Mountains zone	1461200

Table S3. Protected areas within conservation priority areas of *M. moschiferus*

ID	Name	Biogeographic realm	Area (ha)
1	Heilongjiang Beijicun National Nature Reserve	Greater Khingan Mountains zone	137553
2	Heilongjiang Lingfeng National Nature Reserve	Greater Khingan Mountains zone	68373
3	Heilongjiang Nanweng River National Nature Reserve	Greater Khingan Mountains zone	229523
4	Heilongjiang Huzhong National Nature Reserve	Greater Khingan Mountains zone	167213
5	Heilongjiang Pan National Nature Reserve	Greater Khingan Mountains zone	55074
6	Inner Mongolia Hanma National Nature Reserve	Greater Khingan Mountains zone	107348
7	Inner Mongolia Erguna National Nature Reserve	Greater Khingan Mountains zone	124527
8	Heilongjiang Dazhan River Wetland National Nature Reserve	Lesser Khingan Mountains zone	211618
9	Heilongjiang Gongbila River National Nature Reserve	Lesser Khingan Mountains zone	47983
10	Heilongjiang Shengshan National Nature Reserve	Lesser Khingan Mountains zone	60000
11	Heilongjiang Xinqing White-headed Crane National Nature Reserve	Lesser Khingan Mountains zone	62567
12	Heilongjiang Cuibei Wetland National Nature Reserve	Lesser Khingan Mountains zone	27730
13	Heilongjiang Hongxing Wetland National Nature Reserve	Lesser Khingan Mountains zone	111995
14	Heilongjiang Uma River Sable National Nature Reserve	Lesser Khingan Mountains zone	20949
15	Heilongjiang Longxiang National Nature Reserve	Lesser Khingan Mountains zone	31355
16	Heilongjiang Liangshui National Nature Reserve	Lesser Khingan Mountains zone	12133
17	Heilongjiang Liangshui National Nature Reserve	Lesser Khingan Mountains zone	60687
18	Heilongjiang Maolanggou National Nature Reserve	Lesser Khingan Mountains zone	35868
19	Heilongjiang Pingdingshan National Nature Reserve	Lesser Khingan Mountains zone	20241

ID	Name	Biogeographic realm	Area (ha)
20	Heilongjiang Xilian River National Nature Reserve	Lesser Khingan Mountains zone	21570
21	Taipinggou National Nature Reserve	Lesser Khingan Mountains zone	22199
22	Liaoning Baishilazi National Nature Reserve	Changbai Mountains zone	7467
23	Liaoning Laotuziding National Nature Reserve	Changbai Mountains zone	15219
24	Jilin Baishan Musk deer National Nature Reserve	Changbai Mountains zone	21995
25	Jilin Huangnihe National Nature Reserve	Changbai Mountains zone	41583
26	Jilin Ji 'an National Nature Reserve	Changbai Mountains zone	13821.6
27	Jilin Songhua River Sanhu National Nature Reserve	Changbai Mountains zone	115253
28	Jilin Tonghua Shihu National Nature Reserve	Changbai Mountains zone	15200
29	Jilin Zengfengling National Nature Reserve	Changbai Mountains zone	17386
30	Jilin Changbai Mountain National Nature Reserve	Changbai Mountains zone	196465
40	Northeast Tiger and Leopard National Park	Changbai Mountains zone	1461200

Table S4. Characteristics of 69 potential connectivity corridors between 41 core habitats

ID	Core patch		Euclidean Distance (km)	LCP (km)	Resistance
	From_Core	To_Core			
1	1	2	1.37	2.38	7072.65
2	3	5	21.87	28.53	14118.8
3	3	4	33.23	36.78	22375.7
4	4	5	6.35	7.29	5837.4
5	5	6	14.68	18.35	5702.83
6	6	10	105.4	113.23	175112
7	7	9	45.03	50.16	15239.5
8	7	14	52.58	55.6	27341.3
9	7	10	78.02	83.93	27921.6
10	7	12	43.45	46.65	30920.1
11	7	8	67.34	73.05	32379.2
12	7	13	40.05	44.32	34651.4
13	8	10	5.87	6.59	1210.21
14	8	9	10.03	11.55	9150.33
15	9	13	18.37	22.49	9733.16
16	9	10	5.99	10.51	12283.3
17	9	15	41.46	51	22542.2
18	9	14	48.17	53.38	48627.9
19	9	18	72.99	79.98	69290.9
20	10	11	3.51	4.91	1582.71
21	10	15	15.44	17.24	9212.03
22	12	16	3.33	4.06	3228.05
23	12	14	7.32	8.48	4176.52
24	13	14	28.15	31.38	46991.8
25	14	16	20.55	22.84	10840.3
26	14	20	10.42	12.89	14309.5
27	14	18	14.03	15.5	23760.2
28	14	15	70.05	76.5	54806.9
29	15	17	46.58	49.22	29585.6
30	15	18	40.71	44.99	30806.9
31	17	18	8.56	11.49	22694.2
32	18	19	6.24	9.32	8817.82
33	18	20	4.16	5.25	10439.1
34	19	21	1.82	2.87	1646.66
35	19	20	1.62	2.38	4589.16
36	19	22	12.69	14.66	25354.7
37	19	23	13.52	14.92	30828.7
38	20	21	8.19	9.67	19830
39	21	23	17.63	21.45	50942
40	22	23	5.06	6.59	4514.73
41	24	25	6.68	8.13	12195.2
42	25	28	43.85	51.92	95167.2
43	25	26	61.44	71.05	108309
44	26	27	52.34	58.54	103220

ID	Core patch		Euclidean Distance (km)	LCP (km)	Resistance
	From_Core	To_Core			
45	26	28	112.78	148.52	158996
46	26	33	94.77	104.44	160854
47	27	29	14.02	16.6	19360.8
48	28	31	27.4	31.9	17412
49	28	30	26.38	32.51	34935.1
50	28	33	69.44	76.77	103360
51	30	31	14.82	17.44	12686.4
52	30	32	7.44	7.98	16218.5
53	30	33	32.2	36.66	50666.3
54	31	32	10.95	13.38	7451.46
55	31	34	47.07	60.66	51094.3
56	31	35	111.51	124.32	311221
57	32	34	33.59	39.38	41756.6
58	33	34	9.49	11	7024.95
59	34	36	16.36	18.72	10855.8
60	34	35	98.5	105.78	127216
61	35	37	5.58	8.13	11603.6
62	35	36	86.03	92.81	154534
63	37	41	33.81	37.82	68175.9
64	37	39	34.01	37.44	83533.5
65	37	38	44.91	51.23	108462
66	38	39	3.46	5.4	21283.7
67	39	40	3.25	4.91	14410.7
68	39	41	28.57	31.67	76414.7
69	40	41	30.3	37	66853

Reference

1. Zhang, D.D.; Zhu, H.Q.; Ge, Z.Y.; Chang, S.H.; Li, C.; Zhang, X.D. Selection of musk deer winter habitat in Huangnihe Nature Reserve. *Journal of Northwest A&F University (Natural Science Edition)* **2015**, *43*, 15-20, doi:10.13207/j.cnki.jnwafu.2015.06.001.
2. Sun, H.Y.; Li, L.; Yin, Y.X.; Lu, X.D.; Zou, S.C.; Tian, J.L.; Yu, H.W.; Ge, D.N. The Monitoring Research of the Amur Tiger in China by Using a Far Infrared Automatic Camera. *Foresty science and technology* **2015**, *40*, 51-55.
3. Lei, X. Analysis of the Law of on Population Quantity Changes of the Wild Animals *Martes zibellina*, *Moschus moschiferus*, *Tetrao parvirostris* in Ling Feng Nature Reserve. *Forest Investigation Design* **2015**, 77-79, doi:10.3969/j.issn.1673-4505.2015.02.032.
4. Wu, J.P.; Zhang, Y.; Fu, D.F.; Lan, W.X. Comparison of Home Range of Siberian Musk Deer and Coral in Winter. *Journal of Northeast Forestry University* **2008**, *36*, 58-60.
5. Zhang, M.; Zhao, W.S.; Zhao, W.Y.; Li, J.M. Status and administrative measures of Moschus moschiferus resources in Liaoning province. *Liaoning Foresty science and technology* **2005**, 57-59, doi:10.3969/j.issn.1001-1714.2005.03.024.
6. Long, Z.W.; Wang, Y.; Wang, Q.J.; Lan, J.Y.; Piao, M.J.; Zhu, H.Q. Resources Situation and Distribution of Moschus moschiferus in Changbai Mountains in Jilin Province. *Journal of Economic Animal* **2016**, *20*, 207-211.
